# Sensitive and Flexible Polymeric Strain Sensor for Accurate Human Motion Monitoring

**DOI:** 10.3390/s18020418

**Published:** 2018-02-01

**Authors:** Hassan Khan, Amir Razmjou, Majid Ebrahimi Warkiani, Ajay Kottapalli, Mohsen Asadnia

**Affiliations:** 1 School of Engineering, Macquarie University, Sydney 2109, Australia; hassan.khan@students.mq.edu.au; 2 Department of Biotechnology, Faculty of Advanced Sciences and Technologies, University of Isfahan, Isfahan 81746-73441, Iran; amir.razmjouchaharmahali@mq.edu.au; 3 School of Biomedical Engineering, University of Technology Sydney, Sydney 2007, Australia; Majid.Warkiani@uts.edu.au; 4 Center for Environmental Sensing and Modeling (CENSAM) IRG, Singapore-MIT Alliance for Research and Technology (SMART) Centre, 1 Create Way, Singapore 117543, Singapore; ajay@smart.mit.edu

**Keywords:** flexible sensor, human motion detection, PVDF electrospun nanofiber, strain sensors, stretchable sensor, piezoelectric polymers

## Abstract

Flexible electronic devices offer the capability to integrate and adapt with human body. These devices are mountable on surfaces with various shapes, which allow us to attach them to clothes or directly onto the body. This paper suggests a facile fabrication strategy via electrospinning to develop a stretchable, and sensitive poly (vinylidene fluoride) nanofibrous strain sensor for human motion monitoring. A complete characterization on the single PVDF nano fiber has been performed. The charge generated by PVDF electrospun strain sensor changes was employed as a parameter to control the finger motion of the robotic arm. As a proof of concept, we developed a smart glove with five sensors integrated into it to detect the fingers motion and transfer it to a robotic hand. Our results shows that the proposed strain sensors are able to detect tiny motion of fingers and successfully run the robotic hand.

## 1. Introduction

When strain sensors were initially used to detect to measure the level of fatigue in materials. Recently, flexible and wearable strain sensors have found numerous applications in high-end devices [1,2]. By definition, strain sensors are devices that transform mechanical deformation into electrical signals. The potential scope of flexible strain sensors attracts numerous applications given their high reliability, low maintenance, and strain sensing capabilities [2]. Flexible Strain sensors can be used in a variety of industrial, automotive, medical, biomedical, sports, aviation, robotics and consumer electronic applications [2,3]. Some more advanced application of flexible strain sensors are body integrated electronic systems, which can be attached to the skin or clothing to measure precise strain ranging from pulse rate [4,5], heartbeat to bending of joints [6,7,8]. 

When designing a flexible strain sensor, it is necessary to consider the correct fabrication methods and type of materials used to develop a low-cost, surface mountable and sensitive device. A practical strain sensor also requires many specifications including sensitivity, stretchability, flexibility, linearity, durability and response time [2]. In the past, two main categories of strain sensors were developed that are Capacitive and Resistive types, but more recently strain sensors based on piezoelectric materials have been developed [9]. Generally, resistive strain sensors are composed of a strain gauge attached or deposited on a bendable substrate. When a mechanical stress is applied to the sensor, electrical resistance changes as a result of the microstructural fluctuations in its sensing layer [10]. The main component of a capacitive type strain sensors is a dielectric film, which is usually the middle layer between two extended flexible electrodes. Applying a tensile force changes the distance between electrodes, which alter the capacitance in the sensor [11]. All of these types of sensors require substantial amount of power for operation, which often limits their applicability. Piezoelectric flexible strain sensors convert dynamic mechanical deformation into electrical charge due to the piezoelectric properties of the sensing element. Depending on sensor structure and application, sensing element can be sandwiched between electrodes or can be extended from one electrode to another, which activated *d*_31_ or *d*_33_, respectively, where *d* is piezoelectric coefficient [12]. 

In this study, we used Polyvinylidene fluoride (PVDF) to develop a flexible strain sensor for human motion monitoring. It is known that PVDF nano fiber offers the highest piezoelectric coefficient among other polymers. PVDF has been used in a wide range of applications thanks to its flexibility, and many other interesting mechanical and electrical properties [13]. In the early 2000s, an article was published by Sirohi and Chopra [14] that explored behaviour of piezoelectric elements as a strain sensor by charge generated as a product of piezoelectric effect. Later on, in 2007, after the development of PVDF polymer, this polymer became a subject of interest such that A.V. Shirinov and W.K. Schomburg designed a pressure sensor, which was made of PVDF [15]. During the past decade, there has been a significant interest in developing devices using PVDF materials [16,17,18]. In particular, electrospun PVDF nano fibers show a very high piezoelectric coefficient directly after electrospinning allowing us to use them in sensor applications without requiring a further polling step. The sensors can be fabricated into a desired size or shape as required. PVDF fibers show an outstanding mechanical strength, very low acoustic impedance and exhibit a flat frequency response and a broad dynamic response. Having a very low mechanical impedance enables PVDF piezoelectric nano fibers to be electrospun on a surface without a significant change in its mechanical properties. Moreover, PVDF is a close chemical analogue to teflon (PTFE) and, therefore, has a good chemical and moisture resistivity [19]. All of these interesting properties of PVDF nanofibers make it an attractive option for development of sensitive and flexible strain sensors for human motion detection and many other applications.

Our group has previously showed the application of PVDF nano fibers in developing militarized flow sensors [13] and fuel cells [19]. In this study, we present a PVDF nanofiber strain sensor, which demonstrates a good sensitivity for robotic application. Mechanical and piezoelectric properties of a single PVDF nanofiber are characterized. Moreover, a smart glove was developed by integrating the proposed PVDF strain sensors on five fingers of a glove and transferring the finger motion to a robotic hand. The charge generated in strain sensors due to the mechanical deformation of the fingers were detected, which is proportionally related to the position of the fingers. If the fingers bend more, the charge generated in the associated sensor is higher, and vice versa. Specifications such as low-cost, low-weight, flexibility, and electrical and mechanical properties of the piezoelectric device demonstrate the high capability of these devices for developing wearable devices.

## 2. Materials and Methods

**Figure 1:**
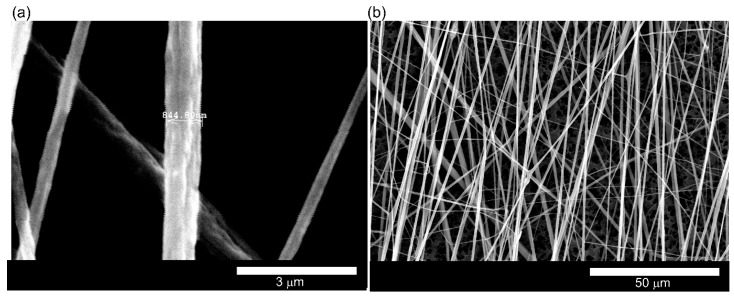
Scanning electron microscope (SEM) images of PVDF electrospun nano fibers (**a**) single nano fiber (**b**) aligned fiber bundle.

Nanofibers were formed from a solution of 1.7 g PVDF powder (MW 534000, Sigma-Aldrich, Sydney Australia) mixed using a magnetic stirrer in 3.7 mL Dimethylformamide (DMF) and 8 mL acetone solvents and heated at 50 °C for about 30 min until a homogeneous solution was achieved. The solution was then transferred into a 10 mL syringe for electrospinning. The feed rate of the precursor polymer, the needle diameter, distance between the needle and the substrate, spinning time and the applied electric field were optimized to achieve fiber with highest piezoelectric coefficient. A direct current (dc) voltage of 12 kV was applied across an 18-gauge syringe needle and a rotating spindle of diameter 100 mm. The polymer solution was dispensed at a feed rate of 5 μL/min. A number of pillar bundle sensors were mounted on the spindle collector, which was positioned 150 mm away from the needle. The spindle rotated a speed of 1500 rpm causing the fibers to stretch as they were deposited on the Polydimethylsiloxane (PDMS) pillars while being electrostatically aligned across the electrode gap. Figure 1 shows the electrospun fibers. After careful process optimization, we achieved fairly uniform fibers with average diameter of about 800 nm.

**Figure 2:**
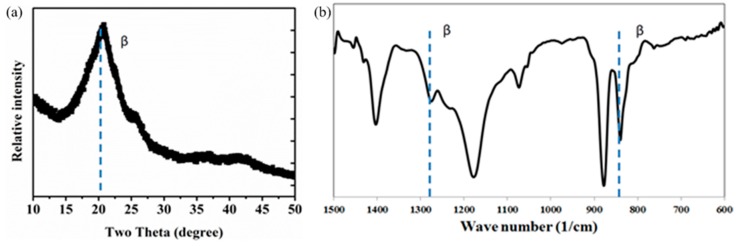
(**a**) XRD and (**b**) FTIR patterns are collected for PVDF nanofibers with a diameter of 800 nm. The peaks reveal characterization of α and β-phases of the nanofibers [13].

To observe the PVDF material phase transformation from Alpha (α) to Beta (β), a comprehensive discussion is presented in our previous paper [13]. Through our electrospinning process, we were able to achieve well-aligned nano fiber with average diameter of 800 nm with high β-phase properties on an aluminum foil substrate. By optimizing the electrospinning time and collector rotation speed, we were also able to obtain single nanofiber on a specially designed substrate. This is important as it allows us to characterize the properties of single PVDF nanofibers. We performed three different analyses (X-ray diffraction (XRD), X-ray diffraction (FTIR) and Raman Analysis) to observe and demonstrate the changing material structure. Figure 2 shows XRD patterns recorded with Siemens 5000 (Simens, Munich, Germany) with Cu Kα radiation (λ = 1.54° A). The conducted test was set in reflection mode under ambient temperature with 2Ø° variation between 10° and 50°, under the scanning speed of 1 min^−1^ and step size of 0.02°. The dominant β-phase structure is shown in Figure 2a. However, absorption of some absorption bands are not apparent in the XRD pattern, which implies a minor presence of α-phase.

FTIR spectra was scanned at 600–1500 cm^−1^, where a total of 32 samples were collected for signal averaging. The bands can be seen in the range of 840 cm^−1^–1280 cm^−1^, which are linked to β-phase, shown in Figure 2b. A unique formation of Beta phase can be seen at 1180 cm^−1^ signifying the separation from alpha phase at 1150 cm^−1^. At 1431 cm^−1^ of the FTIR plot, CH_2_ beta-phase bending mode is also recorded.

**Figure 3:**
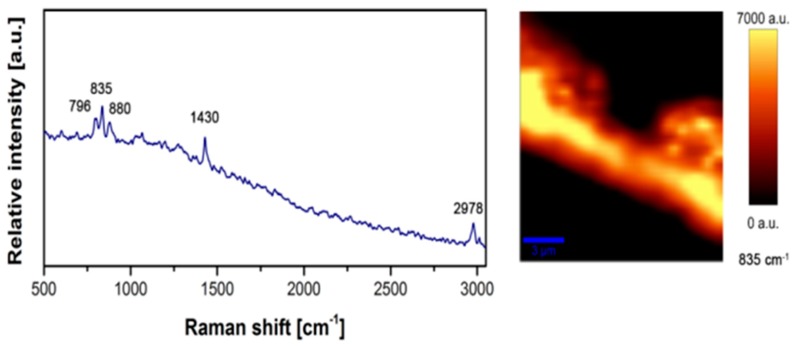
Structural properties of the nano fibers are investigated using Raman spectroscopy. The main peaks on the plot revealed a high concentration of β-phase and therefore denote a high piezoelectricity of the nanofibers [13].

Confocal Raman Microscopy high-resolution imaging technique for characterization of PVDF NFs’ structural properties was implemented. This technique is efficient, as it provides spectroscopic data, which cannot be achieved by IR or XRD [20]. Our setup was equipped with 633 nm wavelength laser, the magnification of 50x to observe Raman shifts at 500–3000 cm^−1^, silicon was used for calibration and our collected spectra data was filtered/smoothened. The results from Raman spectroscopy are illustrated in Figure 3. Thus, a demonstration that our electrospinning process parameter leads to β-phase transformation and enhancement [20].

## 3. Results

### 3.1. Piezoelectric Characterization of a Single PVDF Nanofiber

**Figure 4:**
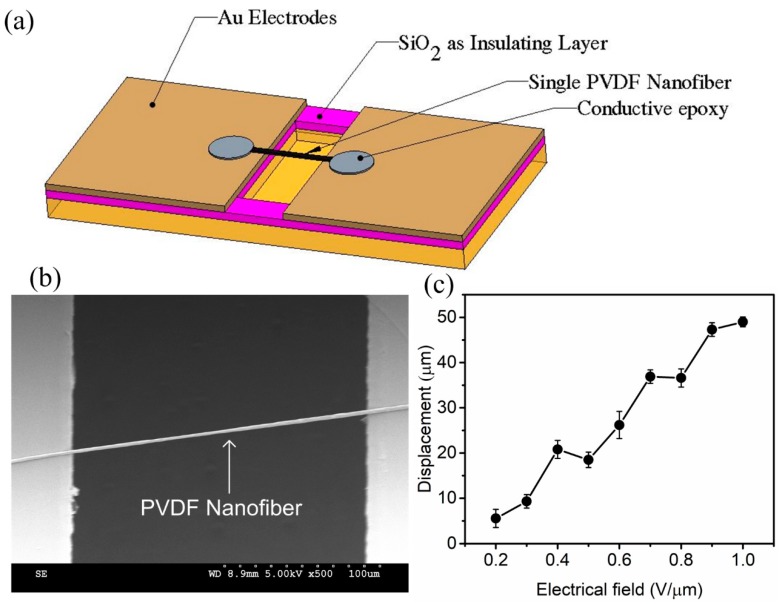
(**a**) Fabricated substrate; (**b**) schematic diagram of the device for measuring the piezoelectric coefficient of a single nanofiber. Insert shows a suspended PVDF nanofiber with a diameter of 800 nm and length of 400 µm; (**c**) shows the displacement of the center of fiber as a function [20].

After successfully electrospinning the sensor, it is critical to observe the piezoelectric coefficient of the single electrospun nanofiber before using it in development of strain sensors. For this purpose, a Microelectromechanical systems (MEMS) substrate was fabricated to hold a single nanofiber for the experiments. Fabrication process commences by cleaning 500 µm of the silicon wafer following by deposition of 1 µm SiO_2_ as an insulated layer using a Plasma-enhanced chemical vapor deposition (PECVD) process. Next, 300 nm Gold was deposited on both sides of the cavity to form into electrodes. Through Hydrofluoric acid (HF) etching, the SiO_2_ layer was removed followed by Deep reactive-ion etching (DRIE) etching to extend the cavity layer to a depth of 300 µm. A schematic of a fabricated MEMS device is shown in Figure 4a. 

After the MEMS substrate fabrication, a single PVDF was electrospun on the substrate and connected to the gold electrodes. To avoid movement and provide a strong connectivity between the fibers and electrode, conductive epoxy was used to fix the fiber on the substrate. Figure 4b shows a SEM image of the single PVDF nanofiber on the MEMS substrate. The sample was then subjected to a different electric field between electrodes ranging from 0^−1^ V/mm and the maximum deformation of nanofiber was recorded under a confocal microscope (Nikon A1R MP + Multiphoton, Nikon, Tohyo, Japan). Figure 4c shows the displacement of the center of fiber as a function of the applied electric field. Our results present a sufficient piezoelectric coefficient d_33_ = −58.7 pm/V for a single NF after five times testing at each electrical field.

### 3.2. Strain Sensor Fabrication and Characterization

**Figure 5:**
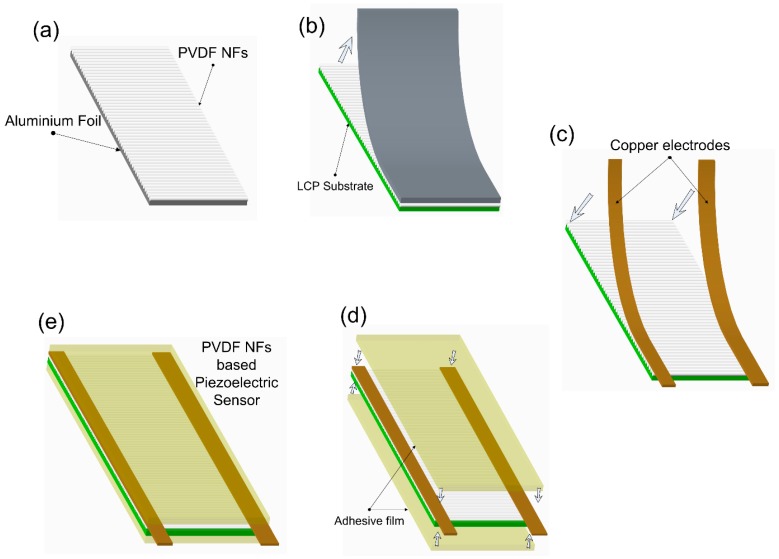
Fabrication process of the high sensitive stretchable strain sensor based on PVDF electrospun nanofiber on a flexible liquid crystal polymer (LCP) substrate. **a**) PVDF nano fibers were collected on an Aluminum foil substrate through far field electrospinning process **b**) Fibers were carefully transferred on a LCP substrate **c**) Copper foil tapes were fixed on the edges of the sensors to form the electrodes **d**) the sensors were laminated by adhesive film to protect the fibers **e**) shows an schematic of the final device.

After mechanical and piezoelectric characterization of single nano fiber, we developed a sufficient and stretchable sensor by using a PVDF nano fiber sensor. The fabrication process in developing the sensor is shown in Figure 5. First, aligned PVDF nanofibers were collected on an aluminum foil substrate. Later on, the fibers were carefully transferred to a flexible liquid crystal polymer (LCP) with 25 µm thickness 10 mm width and 25 mm length. Copper electrode with dimensions 2 mm width and 10 mm length were fixed on two ends of the sensors. The whole sensor was then laminated for the protection of the nanofibers. We used LCP as the substrate for PVDF nanofibers due to its outstanding mechanical properties. Mechanically, LCP has a tensile modulus ranging from 10 to 24 GPa, tensile strength from 125 to 255 MPa and very low moisture absorption (0.02%) and permeability. Chemically, LCP is extremely inert to a wide range of chemicals, including acids and solvents. LCP is fire resistant and produces relatively non-toxic combustion by-products.

### 3.3. Dynamic Pressure Testing Using Dipole Stimulus 

**Figure 6:**
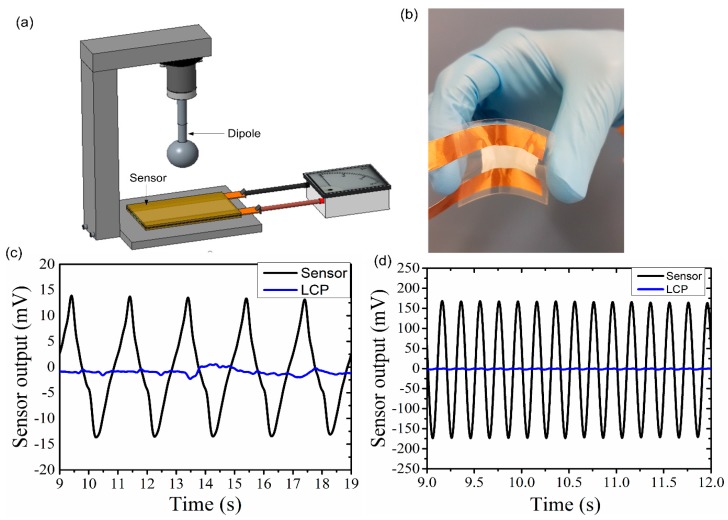
Testing the sensor under low-frequency dynamic loads. (**a**) schematic diagram of the experimental setup. (**b**) image of the flexible PVDF electro spun nanofiber sensor after packaging. (**c**,**d**) experimental result when the vibrating sphere vibrates at a frequency of 0.5 Hz and 5 Hz, respectively at constant amplitude of 250 mVrms.

Since the primary purpose of designing this sensor is to use it for human motion detection, it is critical to ensure the sensors function at very low frequencies (below 2 Hz) in which most of the human motion occurs. A vibrating sphere (Dipole) was used to evaluate the performance of the proposed sensor under dynamic pressure by generating an oscillatory pressure and then the sensor output is observed under various frequencies. The detail of the vibrating sphere oscillator system is described elsewhere [12,21]. Dipole is kept at a distance of 2 mm above the sensor and amplitude of vibration is kept constant (250 mV_rms_), while the frequency changed from 0.5 Hz to 5 Hz. Figure 6a shows the schematic of the experiment. The object that generates the stimulus is a stainless sphere (vibrating sphere) of 8 mm diameter, which is attached to a minishaker (model 410, B & K, Norcross, GA, USA) through a rod of 2 mm diameter. The minishaker was driven by a sinusoidal signal generated by a function generator amplified through a power amplifier (Type 2718, B & K). Data of peak to peak amplitudes of the sensor outputs were recorded using LABVIEW software (version, National Instruments, Austin, TX, USA) as the temperature increases. During the experiments, sensors were directly connected to a data-acquisition card without using any external electrical filters or amplifiers. The experiment was repeated on four different sensors to ensure the repeatability of the results. Figure 6c,d show the sensor output as a function of time for frequency of 0.5 Hz and 5 Hz, respectively. In order to ensure that the voltage generated is actually from PVDF nanofibers and not a result of coupling with dipole, a device made of LCP with the same electrode set-up but with no nanofibers (marked as LCP in Figure 6) was tested under the same experimental conditions as that of the nanofiber sensor. From Figure 6, it is clear that the device without sensing element shows no clear output, indicating no experimental error or coupling in the system.

### 3.4. Finger Bending Experiment

Recently, there has been a lot of interest on human interactive electronics devices that use flexible and wearable devices. The proposed smart glove in this work based on stretchable PVDF nanofibers offers a low-cost, and wearable technology that can be used for fine-motion control in robotics or similar virtual reality platforms where a large strain (ε > 50%) and bending angle (θ > 150) by the movement of the human body need to be detected. 

**Figure 7:**
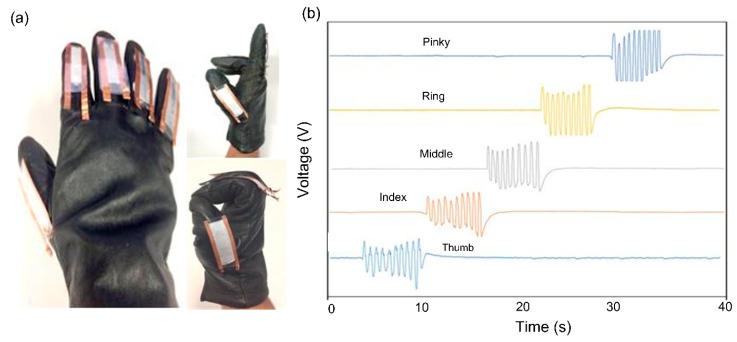
Flexible PVDF electrospun strain sensors for human-motion detection (**a**) photograph of five individual sensors mounted on a smart glove. The states of bending and stretching the glove fingers are also shown; (**b**) sensor output as a function of time for five independent strain sensors when each sensor move at frequency of about 1 Hz.

The performance of the sensors in detecting the movement of the joints in a human hand was examined by implanting five individual sensors on a glove. An overview image of the glove and the sensors in stretching and bending states of the fingers is shown in Figure 7a. Extra care was taken when attaching the sensors to the glove to avoid sliding or unnecessary bending, which may affect the output signal. A National Instruments (National Instruments, Austin, TX, USA) data acquisition (NI-DAQ) system USB-6289 M-series model was integrated into the smart glove to transfer the sensor data to a computer. Figure 7b shows the response behavior of each sensor when its associated finger repeatedly bent and stretched with frequency of about 1 Hz. 

From this experiment, it is evident that the PVDF electrospun sensors can be employed for the accurate motion detection of the human joints due to its excellent bendability and sensitivity. Since the primary idea of developing our sensor was to use it in robotic application, as a proof of concept, the developed smart glove system with five PVDF nanofiber strain sensors was deployed to remotely control a robotic hand. We constructed a robotic hand using micro servo motors that take feedback data and can be controlled via an input signal, which, in this case, would be a signal received from the sensors. Many types of servo motors on the micro servo were selected based on their price, size, and convenience. The voltage change of the sensors was collected via an Arduino card that was customized with our software code. This experiment required five servo motors, one for each finger. To convert charge generated into a signal that can then be fed into servo motors, an Arduino Uno was used as a controller board. Arduino Board is a microcontroller usually used in digital devices, which enables interaction between mechanisms. Similarly, the proposed sensors in smart gloves were then connected to Arduino UNO. Once connected, a voltage divider circuit was established on a breadboard. Bending/straightening of fingers was utilized to control the bionic hand to perform various finger movements such as hold and release. 

**Figure 8:**
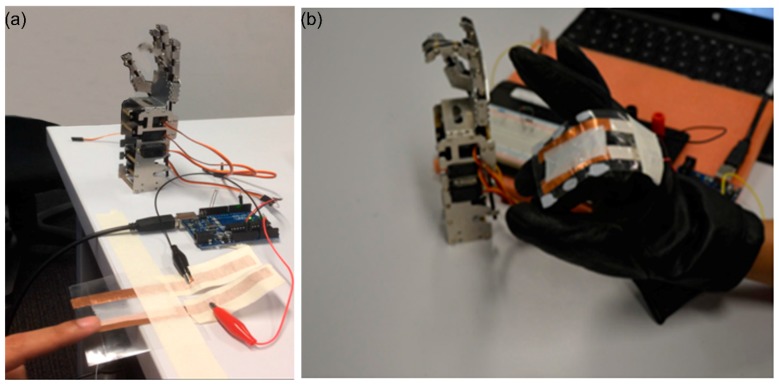
(**a**) experimental setup of using one PVDF electrospun strain sensor connected to a robotic finger; (**b**) experimental setup of mounting five sensors on a glove to detect finger motion and transfer it to the robotic arm (video of experiment is available in the Supplementary Materials).

For example, Figure 8 shows the motion detection for index and middle fingers. As the glove finger bends further, the charge generated in the sensor increases, which, in turn, gives a measurable quantity to the amount of bend in the finger. There are numerous applications for smart gloves, including input gear for entertainment systems, master devices for teleoperated robotic systems, etc. These experiments are a step closer towards piezoelectric strain sensors being the design, fabricated and implemented into a robotic application. Although there were some drawbacks such as restrictions in signal conditioning, mapping hand movement and system stability, these piezoelectrical sensors show tremendous potential for single trigger or flex movement in robotic application at its initial stage.

## 4. Conclusions

In summary, this paper presented the electrospinning process of a PVDF nanofiber and the effect of external parameters on the quality of the nanofibers. A comprehensive characterization on mechanical and piezoelectric properties of a single PVDF nanofiber is done using XRD, FRIT, and Raman spectroscopy studies. We also developed PVDF electrospun nanofiber based strain sensors with good sensitivity with simple and low cost of fabrication process. We have found that the proposed strain sensors have a good response to the bending and joint angle measurement. Finally, a smart glove made of the stretchable strain sensors assembled in each finger was fabricated and used for the real-time motion detection of fingers. As an application, the proposed strain sensors have been used in posture detection and the control of a robotic hand using our smart glove device. From our research and testing, it is clear that our strain sensor devices will help develop new areas of study and research into the applications in flexible, stretchable and wearable electronics due to their excellent performances, especially in human motion detection applications where strain should be accommodated by the strain sensor.

## Supplementary Materials

The following are available online at www.mdpi.com/1424-8220/18/2/418/s1, Video S1: Hand, Table: S2: Thumb.
